# Low Expression of *FFAR2* in Peripheral White Blood Cells May Be a Genetic Marker for Early Diagnosis of Acute Myocardial Infarction

**DOI:** 10.1155/2020/3108124

**Published:** 2020-01-25

**Authors:** Jianjun Ruan, Heyu Meng, Xue Wang, Weiwei Chen, Xiaomin Tian, Fanbo Meng

**Affiliations:** Jilin Provincial Precision Medicine Key Laboratory for Cardiovascular Genetic Diagnosis (Jilin Provincial Engineering Laboratory for Endothelial Function and Genetic Diagnosis of Cardiovascular Disease Jilin Provincial Molecular Biology Research Center for Precision Medicine of Major Cardiovascular Disease Jilin Provincial Cardiovascular Research Institute), Department of Cardiology, China-Japan Union Hospital of Jilin University, Changchun 130033, China

## Abstract

**Objective:**

To find molecular markers for the diagnosis of acute myocardial infarction (AMI), this research further verified the relationship between the expression level of *FFAR2* gene and AMI by expanding the sample size based on the previous gene chip results.

**Methods:**

Peripheral venous leukocytes were collected from 113 patients with AMI and 94 patients with noncoronary artery disease as the experimental group and the control group, respectively. Real-time fluorescence quantitative polymerase chain reaction was used to detect the expression of the *FFAR2* gene. Western blot analysis was applied to detect the relative expression of the *FFAR2* gene at the level of protein. Furthermore, the relationship between gene expression and clinical data was also analyzed and compared.

**Results:**

The level of expression of *FFAR2* gene in peripheral blood of patients with AMI was significantly lower than that of the control group (0.33 [0.04–1.08], 0.62 [0.07–1.86], respectively; *p* < 0.05), which was 0.53 times that of the control group. Western blot results presented that the *FFAR2* protein level in the peripheral blood of the AMI group was lower than that of the control group (0.114; *p*=0.004). Analyzing clinical data of the subjects indicated that the average age of the AMI group was significantly higher than the age of control group (*p* < 0.01). Also, the fasting blood glucose level was higher (*p* < 0.01), and the high-density lipoprotein cholesterol (HDL-C) level was lower (*p*=0.03). The *FFAR2* mRNA level correlated positively with the HDL-C level (*p* < 0.01). Logistic regression analysis suggested that the low expression of the *FFAR2* gene in peripheral blood may be a risk factor for AMI independent of age, family history of diabetes, fasting blood glucose level, and HDL-C level (*p*=0.025). Compared with the high *FFAR2* expression group, the risk of AMI in the low *FFAR2* expression group was 6.308 times higher.

**Conclusion:**

The expression level of the *FFAR2* gene in peripheral blood of patients with AMI was significantly lower than that in the control group. Low expression of the *FFAR2* gene in peripheral blood is an independent risk factor for AMI. Hence, it may also be a potential biomarker to predict AMI.

## 1. Introduction

Acute myocardial infarction (AMI) is a serious consequence of coronary atherosclerotic heart disease [1]. AMI is also one of the cardiovascular diseases with high morbidity and mortality [2,3]. Nearly half of patients with cardiovascular diseases die from AMI [4]. By 2020, cardiovascular disease is expected to be the leading cause of death in both developed and developing countries [5], accounting for 36% of all deaths worldwide [6]. In the United States, the death toll from AMI is more than 2.4 million [7]. According to China's cardiovascular disease report, 11 million patients are suffering from coronary heart disease. The incidence of cardiovascular diseases in China is on the rise, accounting for more than 40% of deaths from diseases.

AMI is a polygenic disease and occurs as a result of interaction between genetic and environmental factors. Therefore, it is very important to find molecular markers for early diagnosis to alert physicians of the possibility of AMI.

Eighty percent of genes in peripheral blood cells are expressed in other tissues. Studies have shown that based on the how different genes in white blood cells were expressed from peripheral blood, gene expression can be used as molecular markers for the diagnosis and prognosis of systemic lupus erythematosus, solid malignant tumors, organ transplantation, and other diseases [8]. It is essential in the prediction of cardiovascular diseases and other complex diseases. The aim of this research was to find the diagnostic markers of AMI from peripheral blood leukocytes.

Previous results of gene chip research showed that *FFAR2* gene expression in peripheral blood was lower than that in the control group. Therefore, this research aims to verify the results of the *FFAR2* gene chip from peripheral blood of a large cohort of patients. Furthermore, the research examined whether the *FFAR2* gene can be used as a molecular marker for the diagnosis of AMI.

## 2. Subjects and Methods

### 2.1. Research Subjects

A total of 113 patients with AMI hospitalized in the Department of Cardiovascular Medicine, China-Japan Union Hospital, Jilin University, from March 2018 through May 2018, were selected as cases. The diagnosis of AMI was based on the global definition of myocardial infarction issued by the European Heart Association in 2017 [9], that is, the presence of definite angiopathy confirmed by coronary angiography and severely narrowed and occluded main coronary arteries (left main coronary artery and right coronary artery, etc.) and main branches (circumflex and anterior descending branches, etc.). The exclusion criteria for AMI were as follows: (1) myocardial infarction secondary to ischemic imbalance; (2) myocardial infarction when serum biochemical markers (troponin and myoglobin) were not available; (3) myocardial infarction associated with percutaneous coronary intervention or stent thrombosis; or (4) coronary artery bypass grafting-related myocardial infarction. Ninety-four patients with noncoronary heart disease were treated as the control group. The criteria for inclusion of subjects in the control group were as follows: hospitalized patients with chest pain, coronary angiography showing degree of coronary artery stenosis less than 50%, no secondary changes of pathological Q-wave, T-wave, and ST-segment in ECG, except acute pneumonia, pleurisy, intercostal neuritis, etc.

Informed consent was obtained from all patients before the collection of test samples and sample information. The study was conducted in accordance with the principles and guidelines laid down in the Declaration of Helsinki. Age, sex, history of smoking, hypertension, and diabetes, levels of fasting blood sugar, serum triglyceride (TG), total cholesterol (TC), high-density lipoprotein cholesterol (HDL-C), and low-density lipoprotein cholesterol (LDL-C) were recorded in detail.

### 2.2. Research Methods

#### 2.2.1. Acquisition of Peripheral Blood Lymphocytes

In the morning, 6 ml of fasting peripheral blood of the study subjects was stored in an EDTA tube at 4°C, and lymphocytes were extracted within 4 hour. The reagent used was peripheral blood lymphocyte separating solution. The details of the steps that were followed are (1) fresh anticoagulant was mixed with an equal volume of 0.9% sodium chloride injection evenly; (2) the above mixture was carefully added to an equal volume of human peripheral blood lymphocyte separating solution, followed by centrifugation for 20 minutes at 3000 r/minute; and (3) after centrifugation, the four layers from top to bottom were plasma, milky white lymphocyte layer, transparent separation layer, and erythrocyte layer. The milky white lymphocyte layer was aspirated for use in subsequent experiments.

#### 2.2.2. Synthesis of cDNA of Peripheral Blood Lymphocytes

(1) The Blood Total RNA Kit (Xinjing Biological Reagent Development Co., Ltd., Hangzhou, China) was used to extract total RNA from lymphocytes. In order to avoid RNA degradation or contamination, the extraction process was carried out in strict accordance with the kit instructions. The quality of RNA solution was detected by polyacrylamide gel electrophoresis. 28S and 18S rRNA bands were visible, and the brightness of the 28S rRNA band was about two times that of the 18S rRNA. The concentration and absorbance of the standard samples were determined by an enzyme-labeling instrument. Reverse transcription was performed after meeting the requirements. (2) According to the instructions of the reverse transcription kit (FastKing gDNA Dispelling RT SuperMix, Tiangen Biochemical Technology Co., Ltd., Beijing, China), reverse transcription of the total RNA that met the requirements of the experiment was carried out, and a consistent concentration of RNA was added to each sample. The DNA samples obtained were stored at −80°C for the subsequent fluorescence, quantitative, polymerase chain reaction detection.

#### 2.2.3. Reverse-Transcriptase Polymerase Chain Reaction (RT-PCR) Detection

After diluting the obtained DNA samples 20 times, the SYBR fluorescence quantitative kit (biochemical fluorescence quantitative kit, Taq qPCR synthetic premix, Shanghai, China) was used for PCR amplification. *GAPDH* was used as internal reference gene and *FFAR2* as target gene. The specificity of amplification conditions was determined by software dissolution curves using the ABI-FAST7500 instrument. The sequence of RT-PCR primers used is shown in Table 1.

#### 2.2.4. Western Blotting

Peripheral white blood cells were collected by radioimmunoprecipitation and centrifuged. The supernatant was then collected and placed in water at 98°C for 10 minutes. Buffer (5x) and 30 *μ*g sample protein were added. The voltage in the laminated gel was set at 60 V. When the protein band was straight and reached the boundary between the stacking gel and the separation gel, the voltage was adjusted to 110 V until the end of electrophoresis. According to the instructions of BDTM semidry ink–absorbing paper, the protein was transferred to polyvinylidene fluoride film and incubated overnight with the primary antibody at 4°C. The secondary antibody was incubated at room temperature for 2 hours and analyzed by a chemiluminescence imaging system.

### 2.3. Statistical Analysis

All the data were analyzed by SPSS 25.0 software. A normality test was used to test the measurement data; $\bar{X}\pm S$ was used to describe data obeying a normal distribution (*p* > 0.1). Two independent samples *t* tests were used to compare the differences between the groups. Median and quartile intervals were used for statistical analysis for the data not obeying a normal distribution (*p* ≤ 0.1). The nonparametric rank sum test of two independent samples was used to compare the differences between the groups. Frequency analysis was used to describe the counting data, and a *χ*^2^ test was used to analyze the differences between the groups. Bivariate logistic regression analysis was used to analyze the risk factors related to AMI. The results were statistically significant with a bilateral *p* ≤ 0.05.

## 3. Results

### 3.1. Baseline Data Analysis

Clinical data of the research subjects showed that there were no significant differences in gender, history of hypertension, smoking history, serum TG level, TC level, and LDL-C level between the AMI group and the control group (*p* > 0.05). However, compared with the patients in the control group, those in the AMI group were significantly older (*p* < 0.01). More people had type 2 diabetes (*p*=0.02). Fasting blood sugar level was significantly higher (*p* < 0.01). HDL-C level was lower (*p*=0.03) (Table 2). The proportion of hypoglycemic drugs used in the AMI group was significantly higher than that in the control group (*p*=0.01) (Table 3).

### 3.2. Analysis of the *FFAR2* Gene

#### 3.2.1. Identification of RT-PCR Products

In this research, the amplification curves of the internal reference and *FFAR2* genes were significantly smooth “S” shaped curves and the dissolution curves had a single peak, without multiple peaks, which showed that the amplified primers had strong specificity, suitable reaction conditions, and no nonspecific amplification.

#### 3.2.2. Analysis of Expression Level of the *FFAR2* Gene

**Figure 1:**
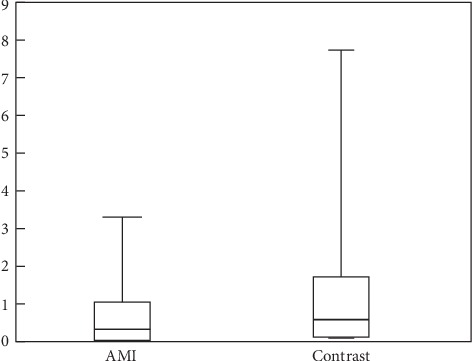
Relative expression of the *FFAR2* gene. AMI = acute myocardial infarction.

**Figure 2:**
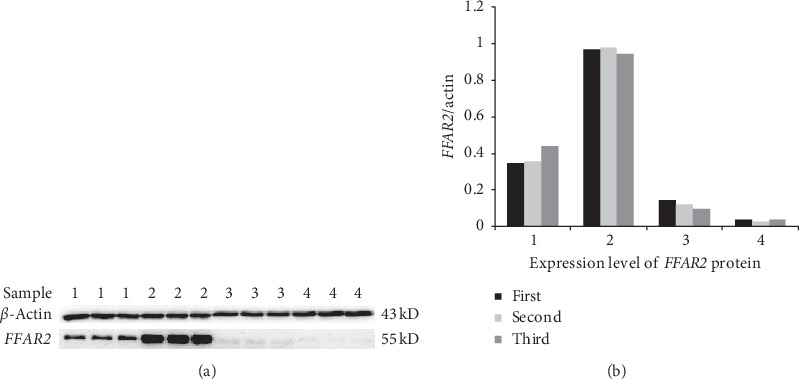
Expression level of *FFAR2* protein. In the control group, the sample numbers are 1 and 2, and in the AMI group, the sample numbers are 3 and 4. AMI = acute myocardial infarction.

RT-PCR was repeated for 3 times for each sample, and the standard deviation was consistent with RT-PCR requirements. Independent sample *t*-test was carried out for the AMI group and the control group, meeting the requirement of *p* < 0.05. The results showed that the relative expression of the *FFAR2* gene in the AMI group (i.e., the 2^−ΔCt^ value quantitatively measured from PCR) was 0.33 (0.04–1.08) and 0.62 (0.07–1.86) in the control group. There was a significant difference between the two groups (*p* < 0.05). The relative expression of the *FFAR2* gene in peripheral blood of patients with AMI was significantly lower (0.53 times) than that in the control group (Figure 1). In this study, beta-actin was taken as the internal reference protein. The protein test was repeated for 3 times for each group to detect the peripheral blood protein level of the research subjects. Western blot results showed that there was no significant difference in the expression of beta-actin between the AMI and control groups, whereas *FFAR2* gene expression was statistically significant between the two groups (*p*=0.004). The expression of the *FFAR2* gene at protein level in the AMI group was 0.114 times of that in the control group (Figures 2(a) and 2(b)).

### 3.3. Correlation Analysis

The baseline data revealed differences between the groups in age, history of diabetes, fasting blood glucose level, and HDL-C. Further analysis was applied to examine whether the relative expression of the *FFAR2* gene correlated with these factors.

All subjects were divided into an elderly group (≥65 years) and a younger group (<65 years), a type 2 diabetes group and a non–type 2 diabetes group, a high fasting blood glucose group (≥5.6 mmol/l) and a normal fasting blood glucose group (<5.6 mmol/l), and a low HDL-C group (<1.04 mmol/l) and the high HDL-C group (≥1.04 mmol/l). The relative expression level of the *FFAR2* gene in each subject was expressed by 2^−ΔCt^. The correlation between the relative expression levels of the *FFAR2* gene in the elderly group and the younger group, between the type 2 diabetes mellitus group and the non–type 2 diabetes mellitus group, between the high fasting blood glucose group and the normal fasting blood glucose group, and between the low HDL-C and high HDL-C groups were statistically analyzed and compared.

The results found no correlation between *FFAR2* gene expression and age (*p*=0.121). The *FFAR2* mRNA level was not correlated with type 2 diabetes mellitus (*p*=0.836), nor with the level of fasting blood sugar (*p*=0.339). However, it correlated with the level of HDL-C (*p* < 0.001). The results are summarized in Table 4.

### 3.4. Logistic Regression Analysis

Based on the cutoff value of relative expression of the *FFAR2* gene, all subjects were divided into a low-expression group (2^−ΔCt^ < 2.850) and a high-expression one (2^−ΔCt^ ≥ 2.850). The sum of sensitivity and specificity is at its peak when using the 2.850 as the cutoff value. According to baseline data, all subjects were divided into a high fasting blood sugar group and a normal fasting blood sugar group, an elderly group and a younger group, a type 2 diabetes mellitus group and a non–type 2 diabetes group, and a low HDL-C group and a high HDL-C group.

**Figure 3:**
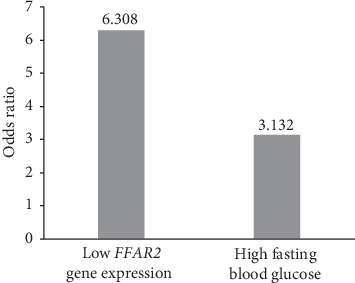
Independent risk factors for AMI. AMI = acute myocardial infarction.

The results were further analyzed using binary logistic regression analysis. The results showed that low expression of the *FFAR2* gene was an independent risk factor for AMI (*p*=0.025) (Table 5). The risk of AMI in the group with low expression of the *FFAR2* gene was 6.308 times higher than that in the group with high expression of *FFAR2* gene. In addition, high fasting blood sugar was an independent risk factor for AMI (*p*=0.008), and high fasting blood sugar increased the risk of AMI to 3.132 times (Figure 3).

## 4. Discussion

This study showed that *FFAR2* gene expression in the peripheral blood of patients with AMI was lower than that in the control group at the level of gene and protein.

*FFAR2*, also known as *GPR43*, is located in a set of standard intron-free genes on chromosome 19q13.1. It encodes a member of the GP40 family of G protein–coupled receptors [10], which belongs to the largest known receptor family [11]. Many molecules, including biogenic amines, amino acids, proteins, fatty acids, lipids, nucleotides, and ions, are activated ligands of G protein–coupled receptors [12]. Short chain fatty acids are mainly produced by intestinal microflora through fermentation of undigested carbohydrates and dietary fibers, which further activates *FFAR2* [10]. The *FFAR2* protein contains seven hydrophobic regions, which are consistent with transmembrane helix (tm). Sequence analysis revealed that *FFAR2* protein belongs to class A of G protein–coupled receptors. The receptors encoded by the *FFAR2* gene contain cysteine residues in the first and second extracellular rings, which may control the structure by forming intramolecular disulfide bonds [13]. *FFAR2*, as a signaling molecule, plays an important role in regulating blood glucose, inflammation, and serum lipid [14]. Abnormal blood glucose, inflammation, and lipid levels increase the risk of AMI in healthy people. The low expression of *FFAR2* may be a potential biomarker to predict the occurrence of AMI by affecting the above pathways.

The results showed that the number of patients with type 2 diabetes mellitus in the AMI group was higher than that in the control group, and the level of fasting blood sugar was higher. Abnormal blood sugar is a recognized risk factor for coronary artery disease [15]. Diabetic patients taking insulin-stimulating drugs show reduced mortality and risk of cardiovascular events [16]. In type 2 diabetes mellitus, the effect of incretin decreased [17,18], and the morbidity and mortality of cardiovascular diseases increased [19]. This was mainly due to the decrease of glucagon-like peptide-1 response associated with diet. Diabetes mellitus can be used as an independent predictor of mortality and new cardiovascular events in hospitalized patients with AMI [20], which may play a role in promoting inflammation in AMI [21].

*FFAR2* and other free fatty acid receptors are considered to be key components of human nutritional sensing mechanism. Studies on these receptors considered them as new therapies for diabetes and other metabolic disorders [22]. The activation of *FFAR2* is coupled with intracellular signals, such as increased IP3 production, increased intracellular Ca^2+^, and the activation of erk1/2 pathway, which contribute to the stimulation of GSI in islets [23]. As a signal molecule of short chain fatty acids, *FFAR2* is coupled with Gαq and Gαi, resulting in the activation of phospholipase C and increase in intracellular calcium levels, or the inhibition of cAMP production by adenylate cyclase, respectively. Thus, it is clear that activation of *FFAR2* contributes to the expansion of pancreatic beta cell clusters and insulin secretion to maintain normal glucose homeostasis [24]. *FFAR2* maintains fasting blood glucose level through the FA signaling pathway [25]. *FFAR2* agonists can be used as a new insulin sensitizer for type 2 diabetes mellitus, with therapeutic potential in this disease [26]. Sodium butyrate, the metabolite of microorganism, can significantly improve the level of *FFAR2*, increase the storage of glycogen, and play a good role in maintaining blood glucose homeostasis, in which *FFAR2*-Akt-Gsk3 pathway may be involved. *FFAR2* is involved in the role of short chain fatty acids in colon cells, adipocytes and immune cells in promoting the secretion of gut hormone, reducing fat decomposition, and regulating immune mediators. The beneficial effect of short chain fatty acids on islets may be secondary to the indirect stimulation and protection of these endocrine cells on islet *β* cells. It can be determined that short chain fatty acids can resist apoptosis of islet cells in a *FFAR2*-dependent manner. When *FFAR2* was reduced or deleted, islet quality and beta cell survival were impaired [14,27], and the conversion of glucagon-like peptide into insulin was reduced [28]. In theory, low expression of the *FFAR2* gene and promotion of blood sugar may be one of the mechanisms of low expression of the *FFAR2* gene promoting AMI. However, the correlation between fasting blood glucose and *FFAR2* gene expression was not observed in this study; However, the baseline data analysis showed that the proportion of hypoglycemic drugs used in the AMI group was higher (*p*=0.01). Hence, we speculated that as more subjects in the hyperglycemic group used hypoglycemic drugs, there was no correlation.

*FFAR2* was originally cloned from white blood cells, and its highest level was detected in neutrophils, monocytes, and other immune cells. The tissue distribution of *FFAR2* indicates that it has a potential role in the activation and differentiation of immune cells [29]. *FFAR2* protein is considered to be the mediator of short chain fatty acids on immune cells. Many studies have confirmed the role of the interaction between *FFAR2* and short chain fatty acids in regulating inflammatory response [30]. *FFAR2* is involved in short chain fatty acids that inhibit histone deacetylase expression and hypermethylation of inflammatory inhibitors [31]. *FFAR2* reduces the production of inflammatory mediators by inhibiting the expression of cytokines and chemokines, and it participates in the regulation of neutrophil activation, affecting inflammatory leukocyte migration [32]. Therefore, decreased expression of the *FFAR2* gene leads to the enhancement of the downstream camp-pka-creb pathway, histone deacetylase (HDAC) overexpression, and inflammation inhibition [31]. It attenuates the inhibition of inflammatory response and increases the activation of circulating inflammatory cytokines, chemokines, and immune cells, which play an important role in AMI.

This research showed that the level of HDL-C in the AMI group was significantly lower than that in the control group (*p*=0.03), and the low expression of the *FFAR2* gene in peripheral blood of the AMI group was associated with a lower level of HDL-C (*p* < 0.001). *FFAR2*, similar to gpr109a (a nicotinic acid receptor), can bind to the gastrointestinal signaling pathway in adipocytes to inhibit fat dissolution, reduce plasma-free fatty acids, and increase the level of HDL-C [33]. In the past few years, epidemiological studies have shown that low concentrations of HDL-C are associated with increased risk of coronary artery disease and cardiovascular events [34]. As an important component of lipid metabolism disorders, abnormal level of HDL-C has increasingly attracted attention. A large number of experiments have proved that HDL-C has a protective effect on atherosclerosis regardless of sex and race. It mainly plays an antiatherosclerotic role by reversing the transport of cholesterol from peripheral tissues. In addition, HDL-C may have antioxidant and anti-inflammatory effects and inhibit cytokine-induced expression of transduced endothelial cell adhesion molecules, vascular cell adhesion molecule-1, and intercellular adhesion molecule-1, and it can also inhibit thrombosis [35]. The relatively low expression of the *FFAR2* gene, which decreases HDL-C level, is one of the mechanisms by which *FFAR2* promotes AMI.

Logistic regression analysis showed that low expression of the *FFAR2* gene in peripheral blood was a risk factor for AMI, independent of age, history of diabetes mellitus, fasting blood glucose level, and HDL-C level (*p*=0.025). Compared with high expression of the *FFAR2* gene, low expression of the *FFAR2* gene increased the risk of AMI to 6.308 times. Theoretically, the low expression of *FFAR2* may be related to the high fasting blood glucose, but the above correlation was not seen in this research. It may be further verified by expanding the sample size due to the use of hypoglycemic drugs in the research subjects. At the same time, high fasting blood glucose level is an independent risk factor for AMI. Compared with normal fasting blood glucose level, the risk of AMI increased to 3.132 times.

## 5. Conclusion

The expression level of the *FFAR2* gene in peripheral blood of patients with AMI was significantly lower than that in the control group. Low expression of the *FFAR2* gene in peripheral blood is an independent risk factor for AMI. One of the mechanisms may be that low expression of the *FFAR2* gene reduces the level of HDL-C and promotes the occurrence of AMI. Low expression of the *FFAR2* gene in peripheral blood may be a potential biomarker in predicting the risk of AMI.

## Figures and Tables

**Table 1:** RT-PCR primer sequence.

Genes		Genes primer sequence (5′–3′)
*FFAR2*	F^a^	CTTCGGACCTTACAACGTGTC
	R^b^	CTGAACACCACGCTATTGAC

*GAPDH*	F^a^	TGTGGGCATCAATGGATTTGG
	R^b^	ACACCATGTATTCCGGGTCAAT

F^a^: upstream primers. R^b^: downstream primers. RT-PCR = reverse-transcriptase polymerase chain reaction.

**Table 2:** Baseline data (which showed no differences between the test and the control groups).

Data category	AMI group (*n* = 113)	Controls (*n* = 94)	*t*/*x*^2^/*z*	*p* value
Gender				
Male (%)	80 (70.80)	46 (48.94)		
Female (%)	33 (29.20)	48 (51.06)		
Hypertension (%)	55 (48.67)	41 (43.62)	0.53	0.47
Smoking (%)	52 (46.02)	40 (42.55)	1.58	0.66
TG (mmol/l)	1.57 (1.12–2.50)	1.29 (1.03–1.97)	−1.81	0.07
TC (mmol/l)	4.47 ± 1.26	4.57 (3.81–5.36)	−0.40	0.69
LDL-C (mmol/l)	2.99 ± 0.98	2.92 ± 0.77	0.51	0.61

AMI = acute myocardial infarction; LDL-C = low-density lipoprotein cholesterol; TC = total cholesterol; TG = triglyceride.

**Table 3:** Baseline data (which showed differences between the test and the control groups).

Data category	AMI group (*n* = 113)	Control (*n* = 94)	*t*/*x*^2^/*z*	*p* value
Age (years)	64.10 ± 11.23	57.88 ± 10.42	4.10	0.00
Type 2 diabetes mellitus (%)	26 (29.89)	12 (15.38)	5.07	0.02
Fasting blood glucose (mmol/l)	6.56 (5.32–9.43)	5.39 (5.03–6.18)	−4.24	0.00
HDL-C (mmol/l)	0.95 (0.81–1.13)	1.05 (0.92–1.24)	−2.24	0.03
Hypoglycemic drug (%)	25 (22.12)	8 (8.51)	7.695	0.01

AMI = acute myocardial infarction; HDL-C = high-density lipoprotein cholesterol.

**Table 4:** Correlation analysis of *FFAR2* mRNA level with age, history of diabetes, fasting blood glucose, and HDL-C level.

Group	*N*	Relative expression of *FFAR2*	*Z*	*p* value
Younger age	121	0.50 (0.06–1.51)		
Older age	86	0.34 (0.03–1.02)	−1.552	0.121
Type 2 diabetes mellitus-free group	126	0.24 (0.02–1.13)		
Type 2 diabetes mellitus group	38	0.26 (0.05–0.86)	−0.207	0.836
High fasting blood glucose group	71	0.39 (0.02–1.45)		
Normal fasting blood glucose group	89	0.23 (0.02–1.08)	−0.956	0.339
Low HDL-C group	103	0.21 (0.02–1.02)		
High HDL-C group	83	0.70 (0.15–1.79)	−3.508	0.000

HDL-C = high-density lipoprotein cholesterol.

**Table 5:** Logistic regression analyses of independent risk factors for AMI.

	*B*	Standard variation	Wald	Degree of freedom	*p* value	OR	95% CI
Low *FFAR2* gene expression	1.842	0.824	5.001	1	0.025	6.308	1.256–31.694
High fasting blood glucose group	1.142	0.428	7.115	1	0.008	3.132	1.354–7.248
Older age	0.610	0.405	2.264	1	0.132	1.840	0.832–4.069
Type 2 diabetes mellitus	0.593	0.528	1.262	1	0.261	1.810	0.643–5.093
Low HDL-C group	0.277	0.412	0.452	1	0.502	1.319	0.588–2.955

AMI = acute myocardial infarction; CI = confidence interval; HDL-C = high-density lipoprotein cholesterol; OR = odds ratio.

## Data Availability

The data used to support the findings of this study have been deposited in the Figshare repository.

## Supplementary Materials

Supplementary Materials Figure S1: RT-PCR curve. (a) RT-PCR amplification curve of the *FFAR2* gene. (b) RT-PCR dissolution curve of the *FFAR2* gene.
