# Supplementary material for: Low Expression of FFAR2 in Peripheral White Blood Cells May Be a Genetic Marker for Early Diagnosis of Acute Myocardial Infarction
Source: Cardiol Res Pract. 2020 Jan 25;2020:3108124. doi: 10.1155/2020/3108124 (PMC7204345; doi:10.1155/2020/3108124)
Supplement: Supplementary Materials — Figure S1: RT-PCR curve. (a) RT-PCR amplification curve of the FFAR2 gene. (b) RT-PCR dissolution curve of the FFAR2 gene. [file 3108124.f1.pdf]

Figure 1 . RT-PCR curve. A: RT-PCR amplification curve of the *FFAR2* gene; B: RT-PCR dissolution curve of the *FFAR2* gene

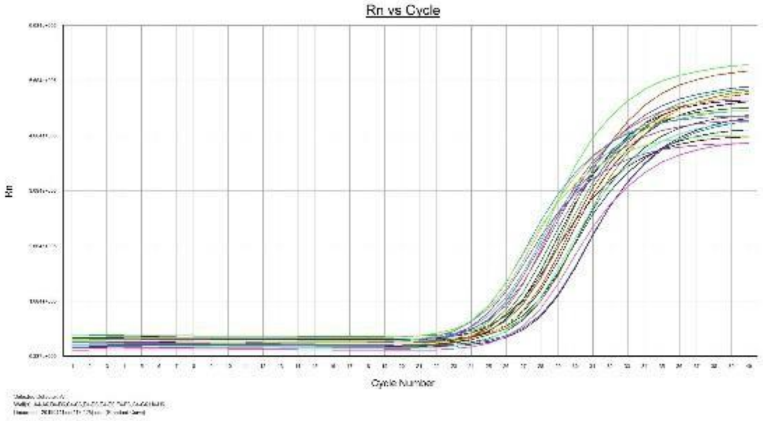

A: RT-PCR amplification curve of the *FFAR2* gene

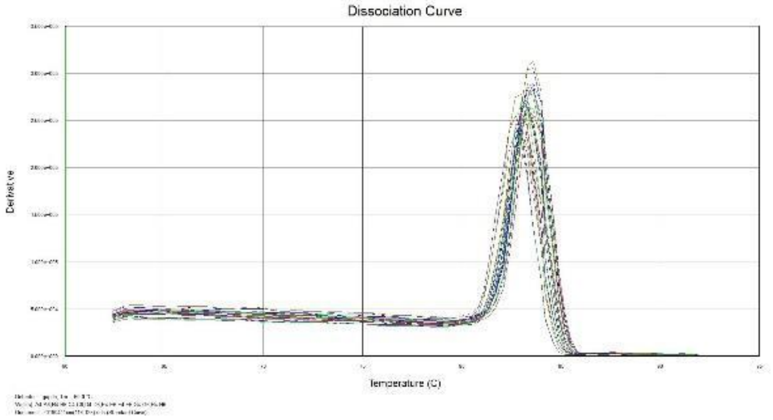

B: RT-PCR dissolution curve of the *FFAR2* gene
